# An effective prediction model based on XGBoost for the 12-month recurrence of AF patients after RFA

**DOI:** 10.1186/s12872-023-03599-9

**Published:** 2023-11-16

**Authors:** ShiKun Sun, Li Wang, Jia Lin, YouFen Sun, ChangSheng Ma

**Affiliations:** 1 The First Affiliated Hospital of Soochow University, Suzhou, 215006 China; 2 The Shengcheng Street Health Center, Shouguang, 262700 China

**Keywords:** Atrial fibrillation, Radiofrequency ablation, Recurrence, Left atrial appendage, XGBoost

## Abstract

**Background:**

Atrial fibrillation (AF) is a common heart rhythm disorder that can lead to complications such as stroke and heart failure. Radiofrequency ablation (RFA) is a procedure used to treat AF, but it is not always successful in maintaining a normal heart rhythm. This study aimed to construct a clinical prediction model based on extreme gradient boosting (XGBoost) for AF recurrence 12 months after ablation.

**Methods:**

The 27-dimensional data of 359 patients with AF undergoing RFA in the First Affiliated Hospital of Soochow University from October 2018 to November 2021 were retrospectively analysed. We adopted the logistic regression, support vector machine (SVM), random forest (RF) and XGBoost methods to conduct the experiment. To evaluate the performance of the prediction, we used the area under the receiver operating characteristic curve (AUC), the area under the precision-recall curve (AP), and calibration curves of both the training and testing sets. Finally, Shapley additive explanations (SHAP) were utilized to explain the significance of the variables.

**Results:**

Of the 27-dimensional variables, ejection fraction (EF) of the left atrial appendage (LAA), N-terminal probrain natriuretic peptide (NT-proBNP), global peak longitudinal strain of the LAA (LAAGPLS), left atrial diameter (LAD), diabetes mellitus (DM) history, and female sex had a significant role in the predictive model. The experimental results demonstrated that XGBoost exhibited the best performance among these methods, and the accuracy, specificity, sensitivity, precision and F1 score (a measure of test accuracy) of XGBoost were 86.1%, 89.7%, 71.4%, 62.5% and 0.67, respectively. In addition, SHAP analysis also proved that the 6 parameters were decisive for the effect of the XGBoost-based prediction model.

**Conclusions:**

We proposed an effective model based on XGBoost that can be used to predict the recurrence of AF patients after RFA. This prediction result can guide treatment decisions and help to optimize the management of AF.

**Supplementary Information:**

The online version contains supplementary material available at 10.1186/s12872-023-03599-9.

## Introduction

Atrial fibrillation (AF) is a common heart rhythm disorder that affects millions of people worldwide. It is characterized by irregular and rapid electrical activity in the upper chambers (atria) of the heart, leading to an irregular heartbeat. The causes of AF are complex and multifactorial. Risk factors for AF include advanced age, hypertension, heart disease, diabetes, obesity, and a family history of the condition [1–3]. Certain lifestyle factors, such as excessive alcohol consumption, smoking, and lack of physical activity, can also increase the risk of developing AF. AF can have serious consequences, including an increased risk of stroke and heart failure. The irregular heartbeat can cause blood to pool in the heart, increasing the risk of blood clot formation that can lead to a stroke. The risk of stroke in people with AF is five times higher than that in those without AF [1]. The diagnosis of AF is typically made using electrocardiography (ECG) or other cardiac monitoring techniques [4, 5]. Treatment for AF includes medication to control the heart rate and rhythm, anticoagulation therapy to reduce the risk of stroke, and sometimes procedures such as cardioversion or ablation to restore normal heart rhythm [6].

Radiofrequency ablation (RFA) is a procedure used to treat AF. During RFA, a catheter with an electrode at its tip is guided into the heart through a vein in the groin or neck. The electrode emits high-frequency radio waves that create heat and isolate the abnormal heart tissue that causes irregular heartbeats [7]. While RFA has been found to be effective in treating AF, some patients experience recurrence of AF after the procedure. Studies have shown that the 12-month recurrence rate of AF after RFA is approximately 15–30% [2, 8]. However, the exact rate may vary depending on several factors, including patient characteristics, the extent of AF, and the techniques used during RFA. Several factors have been identified as predictors of the 12-month recurrence rate of AF after RFA. These include a larger left atrial size, a longer duration of AF, and the presence of underlying heart disease [9, 10]. Additionally, inadequate lesion formation during RFA or incomplete pulmonary vein isolation, the pulmonary vein being a common target during the procedure, can also increase the risk of 12-month recurrence of AF [11]. Patients who experience recurrence of AF after RFA may require further treatment, such as repeat RFA or medical therapy [12–14]. It is essential to closely monitor patients after RFA to detect those who experience recurrence of AF and provide appropriate treatment promptly.

The anatomical substrate of AF patients is mostly derived from the left atrium. Substantial studies have shown a relationship between the anatomy and function of the left atrium and the recurrence of AF after RFA [15, 16]. In addition, as an accessory structure of the left atrium, the left atrial appendage (LAA) is more sensitive to changes in left atrial function, and its role in predicting recurrence after RFA has been reported [17, 18]. Strain imaging of the LAA can reflect the myocardial deformation of the latter, so it can accurately reflect the function of the LAA [19]. However, due to limitations in sample size and method of measurement, the few studies on the relationship between LAA strain and recurrence of AF did not reach a positive outcome [20, 21].

In this study, we used left atrial parameters, optimized LAA indicators combined with clinical demographics and laboratory indicators to construct clinical prediction models based on the XGBoost method for AF recurrence to accurately identify patients prone to recurrence after RFA and implement individualized treatment among AF patients.

Machine learning algorithms are an important branch of artificial intelligence that can automatically learn from data and be used to better explore data information, so they present high application value in prediction in clinical settings [22, 23]. In this study, we used four high-performance algorithms to build predictive models, including logistic regression, support vector machine (SVM), random forest (RF) and extreme gradient boosting (XGBoost), to identify recurrence after RFA among AF patients.

## Methods

### Study population

The Ethics Committee of The First Affiliated Hospital of Soochow University approved the study (No.: 184/2022), and the study conformed to the Declaration of Helsinki (2013 Revision). A total of 392 AF patients who underwent RFA at the First Affiliated Hospital of Soochow University from October 2018 to November 2021 were included in the study. The inclusion and exclusion criteria are presented in Table 1. All patients underwent routine echocardiography and 24-hour ECG in our hospital three months after the procedure to determine the negative remodelling of the left atrium and the presence of atrial arrhythmia episodes. If palpitation or other symptoms occurred, patients were recommended to the nearest hospital and completed a 24-hour ECG as soon as possible. AF recurrence was defined as an episode of atrial arrhythmias persisting for at least 30 s in any way after 3 months. All subjects were followed up for 12 months.

**Table 1:** The inclusion and exclusion criteria

Inclusion criteria	Exclusion criteria
diagnosis of AF	history of myocardial infarction
a minimum age of 18	valvular heart disease
	primary cardiomyopathies
	congenital heart disease
	poor-quality oesophageal ultrasound images
	previously undergone ablation for AF
	underwent or about to undergo occlusion of the left atrial appendage

### Data collection and analysis

All patients’ baseline data were collected from their electronic medical records (EMR), including age, sex, body mass index (BMI), smoking and drinking history, AF classification, hypertension history, diabetes mellitus (DM) history, neutrophil-to-lymphocyte ratio (NLR), haemoglobin concentration, N-terminal probrain natriuretic peptide (NT-proBNP), estimated glomerular filtration rate (eGFR), high sensitivity C-reactive protein (hs-CRP), blood plasma albumin (ALB), and D-dimer levels. In our study, drinking history was defined as moderate or higher drinking, involving more than 8 drinks per week. Twenty-seven-dimensional data (Table 2) were included in the statistical analysis. Variables with a normally distributed or non-normal distribution are expressed as the means ± standard deviations or medians (quartiles), respectively. Categorical variables were expressed as counts (proportion). Numerical differences between two groups were assessed by the Chi-square test or Fisher’s exact test for categorical variables, while the t test and Kruskal–Wallis H test or Mann–Whitney U test were used for continuous variables. The threshold for significance was P = 0.05.

First, parameters with significant differences were initially selected through the T test, Kruskal–Wallis H test or Mann–Whitney U test. Second, considering the collinearity between variables, we further screened the parameters with significant differences from the selected parameters by the backwards stepwise regression method based on the Akaike information criterion (AIC). We also introduced left atrial appendage ejection fraction (LAAEF) as an additional predictor of AF recurrence according to the latest research [24, 25]. Considering that logistic regression is a linear model of the four models, we conducted a collinearity test on the six variables, and the result showed that VIF < 5, so there was no collinearity among the six variables that were finally entered into the models. All data analyses were conducted using Python, Version 3.8.8.

**Table 2:** Basic clinical data of all patients

Variables	Recurrence (n = 61)	Nonrecurrence (n = 298)	P value
**Demographics**			
Age (years), median (IQR)	67 (63, 73)	65 (58, 69)	< 0.01
Sex (female,%)	36 (59.0)	115 (38.6)	< 0.01
BMI (kg/m2, Mean ± SD)	25.3 ± 3.5	24.6 ± 3.0	0.12
**Medical history**			
Smoking (%)	10 (16.4)	49 (16.4)	0.99
Drinking (%)	5 (8.2)	50 (16.8)	0.09
Persistent AF (%)	49 (80.3)	154 (51.7)	< 0.01
**Comorbidities**			
Hypertension (%)	41 (67.2)	177 (59.4)	0.26
Diabetes mellitus (%)	15 (24.6)	31 (10.4)	< 0.01
**Laboratory test, median (IQR)**			
NLR	1.9 (1.4, 2.6)	1.8 (1.3, 2.3)	0.24
Haemoglobin (g/l)	141.0 (128.0, 153.0)	141.0 (128.0, 151.0)	0.64
NT-proBNP (pg/ml)	949.2 (368.5, 1964.0)	368.7 (124.8, 853.3)	< 0.01
eGFR (ml/min/1.73 m^2^,)	98.1 (79.6, 112.2)	98.4 (82.9, 114.0)	0.38
hs-CRP (mg/l)	2.84 (0.9, 5.3)	1.2 (0.6, 2.8)	< 0.01
ALB (g/l)	37.8 (35.5, 39.6)	40.0 (37.7, 42.5)	< 0.01
D-dimer (ug/ml)	0.3 (0.2, 0.7)	0.2 (0.2, 0.3)	0.01
**Echocardiography, median (IQR)**			
LAD (mm)	49.1 ± 5.3	43.7 ± 5.3	< 0.01
RAD (mm)	43.0 (38.0, 47.0)	39.0 (36.0, 43.0)	< 0.01
RVD (mm)	35.0 (32.0, 38.0)	34.0 (32.0, 37.0)	0.20
IVSth (mm)	10.0 (9.0, 10.0)	9.0 (9.0, 10.0)	0.25
LVDD (mm)	50.0 (46.0, 54.0)	49.0 (46.0, 52.0)	0.08
LVEF (%)	57.0 (46.0, 61.0)	61.0 (58.0, 65.0)	< 0.01
E (cm/s)	95.0 (81.0, 109.0)	83.0 (65.0, 101.0)	< 0.01
E/e’	10.9 (8.3, 14.2)	9.2 (7.4, 11.7)	< 0.01
EDT (ms)	173.0 (135.0, 206.0)	187.0 (155.0, 226.0)	< 0.01
**LAA, median (IQR)**			
LAAEF (%)	30.0 (21.0, 47.0)	65.0 (43.0, 90.0)	< 0.01
LAAD (mm)	20.0 (27.0, 53.0)	19.0 (17.0, 21.0)	0.06
LAAGPLS	6.1 (4.0, 8.9)	13.1 (9.1, 18.8)	< 0.01

**BMI, body mass index; NLR, neutrophil-to-lymphocyte ratio; NT-proBNP, N-terminal pro-B-type natriuretic peptide; eGFR, estimated glomerular filtration rate; hs-CRP, hypersensitive C-reactive protein; ALB, albumin; LAD, left atrial diameter; RAD, right atrial diameter; RVD, right ventricular diameter; IVSth, interventricular septum thickness; LVDD, left ventricle end-diastolic diameter; LVEF, left ventricular ejection fraction; E, peak mitral valve flow velocity during early ventricular diastole; e’, peak mitral ring motion velocity during early ventricular diastole; E/e’, ratio of E to e’; EDT, E-wave deceleration time; LAAEF, left atrial appendage ejection fraction; LAAD, left atrial appendage diameter; LAAGPLS, left atrial appendage global peak longitudinal strain**.

### Model development and validation

**Fig. 1:**
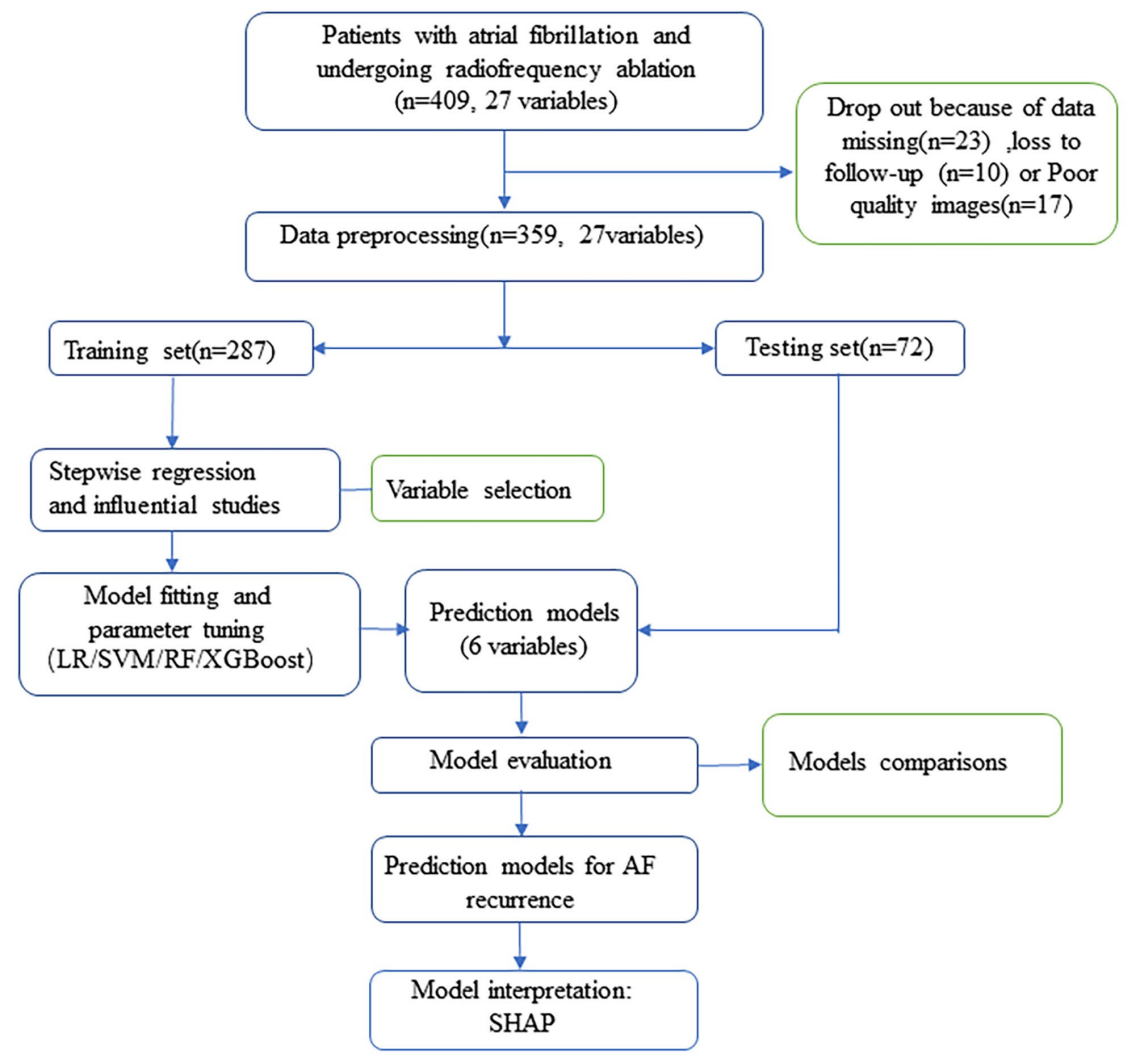
Flow chart of study population selection and model construction

All data were divided into a training set and a testing set at a ratio of 8:2. Four machine learning algorithms were utilized to construct the prediction model, including logistic regression, SVM, RF and XGBoost. Grid searches were used to adjust the parameters. In this study, we utilized 5-fold cross-validation during the model construction process. The combined area under the receiver operating characteristic curve (AUC) was used to evaluate the accuracy and calibration performance and to select the optimal model for predicting the 12-month recurrence of AF after RFA. Moreover, in this study, we also applied the SHapley Additive exPlanations (SHAP) algorithm to fully demonstrate the marginal contribution of each variable in the optimal model. The data collection, model construction, and evaluation processes are shown in Fig. 1.

## Results

### Comparison of clinical data between the two groups

**Fig. 2:**
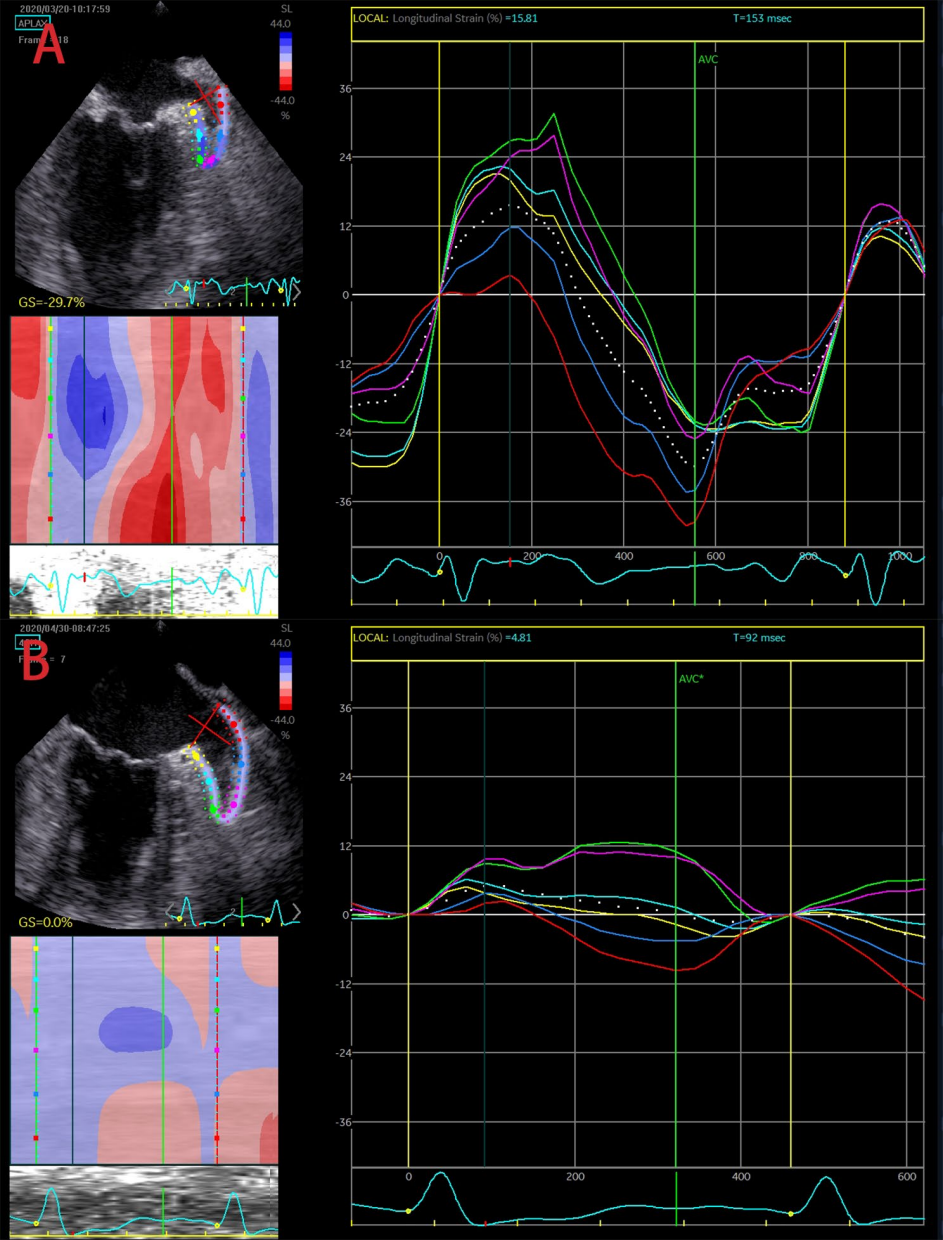
Echocardiographic speckle tracking imaging of LAA strain mapping in AF patients. (**A**) Nonrecurrence at 12 months after RFA; (**B**) recurrence at 12 months after RFA

After 17 patients were excluded due to poor-quality oesophageal ultrasound images, a total of 392 patients diagnosed with AF were included in this study. Thirty-three patients were excluded from the study due to missing data or loss of follow-up. A total of 359 AF patients were eventually enrolled in the analysis, with 61 patients in the recurrence group and 298 in the nonrecurrence group according to their recurrence status at their 12-month follow-up. Comparison of demographics, laboratory data, echocardiography and oesophageal echocardiography results between the two groups showed significant differences in age, sex, presence of persistent AF, diabetes history, NT-proBNP, hs-CRP, ALB, D-dimer, left atrial diameter (LAD), right atrial diameter (RAD), left ventricular ejection fraction (LVEF), peak mitral valve flow velocity during early ventricular diastole (E), peak mitral ring motion velocity during early ventricular diastole (e’), ratio of E to E’ (E/e’), E deceleration time (EDT), left atrial appendage ejection fraction (LAAEF) and left atrial appendage global peak longitudinal strain (LAAGPLS) as shown in Table 2. Representative LAA strain images from patients in the two groups are shown in Fig. 2.

### Variable selection

We conducted a multivariate backwards stepwise logistic regression analysis to identify factors associated with recurrence of AF after univariate regression (Table 3). Five variables, female sex (OR: 2.104, 95% CI: 1.018, 4.349, P = 0.05), DM (OR: 2.362, 95% CI: 0.945, 5.903, P = 0.07), NT-proBNP (OR: 1.000, 95% CI: 1.000, 1.000, P = 0.03), LAD (OR: 1.139, 95% CI: 1.057, 1.228, P < 0.01), and LAAGPLS (OR: 0.763, 95% CI: 0.690, 0.844, P < 0.01), were screened by stepwise regression (P < 0.1) as shown in Table 4. The role of reduced LAAEF in predicting AF recurrence after RFA has been shown in recent studies [24, 25]. Therefore, we also incorporated LAAEF into our prediction model.

**Table 3:** The results of univariate regression

variables	Univariate analysis		
	OR	95% CI	P
Age (years)	1.054	1.019, 1.090	< 0.01
Female	2.291	1.308, 4.016	< 0.01
Persistent AF	3.818	1.952, 7.469	< 0.01
DM	2.871	1.436, 5.739	< 0.01
NT-proBNP (pg/ml)	1.001	1.000, 1.001	< 0.01
hs-CRP (mg/l)	1.098	1.033, 1.166	< 0.01
ALB (g/l)	0.807	0.738, 0.882	< 0.01
D-dimer (ug/ml)	1.156	1.021 ,1.449	0.02
LAD (mm)	1.205	1.136, 1.277	< 0.01
RAD (mm)	1.078	1.028, 1.131	< 0.01
LVEF (%)	0.925	0.900, 0.951	< 0.01
E (cm/s)	1.020	1.008, 1.032	< 0.01
E/e’	1.105	1.041, 1.173	< 0.01
EDT (ms)	0.992	0.986, 0.998	0.01
LAAEF (%)	0.954	0.940, 0.967	< 0.01
LAAGPLS	0.730	0.666, 0.800	< 0.01

**Table 4:** The results of stepwise regression

variables	Stepwise regression		
	OR	95% CI	P
Female	2.104	1.018, 4.349	0.045
DM	2.362	0.945, 5.903	0.07
NT-proBNP (pg/ml)	1.000	1.000, 1.000	0.03
LAD (mm)	1.139	1.057, 1.228	< 0.01
LAAGPLS	0.763	0.690, 0.844	< 0.01

### Model construction and evaluation

**Fig. 3:**
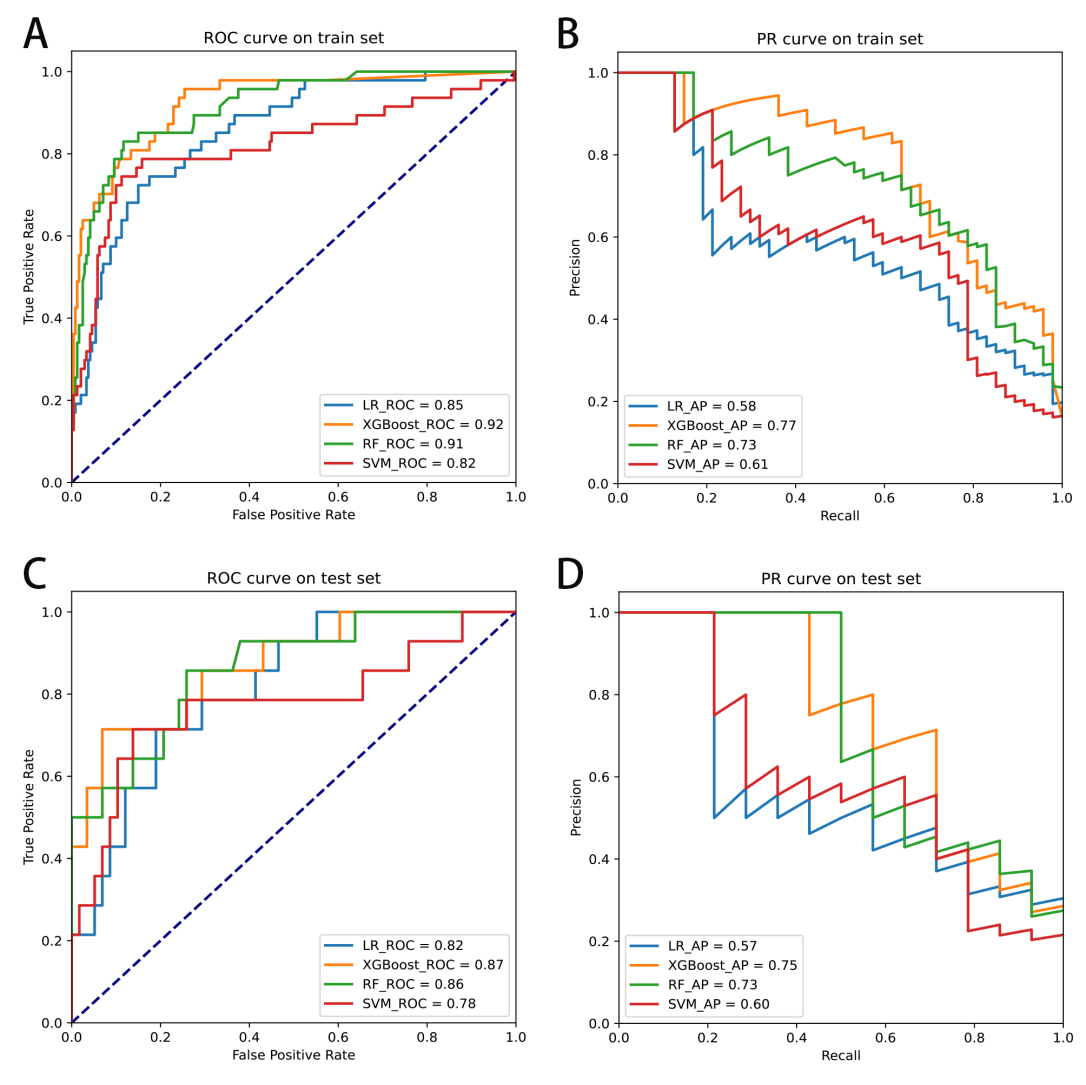
(**A**) ROC curve of the training set in the logistic regression, SVM, RF and XGBoost models; a larger AUC indicates a stronger discriminatory ability; (**B**) PR curve of the training set in the logistic regression, SVM, RF and XGBoost models; a larger AP indicates a better identification ability of 12-month recurrence in the training set data; (**C**) ROC curve of the testing set in the logistic regression, SVM, RF and XGBoost models; (**D**) PR curve of the testing set in the logistic regression, SVM, RF and XGBoost models

**Fig. 4:**
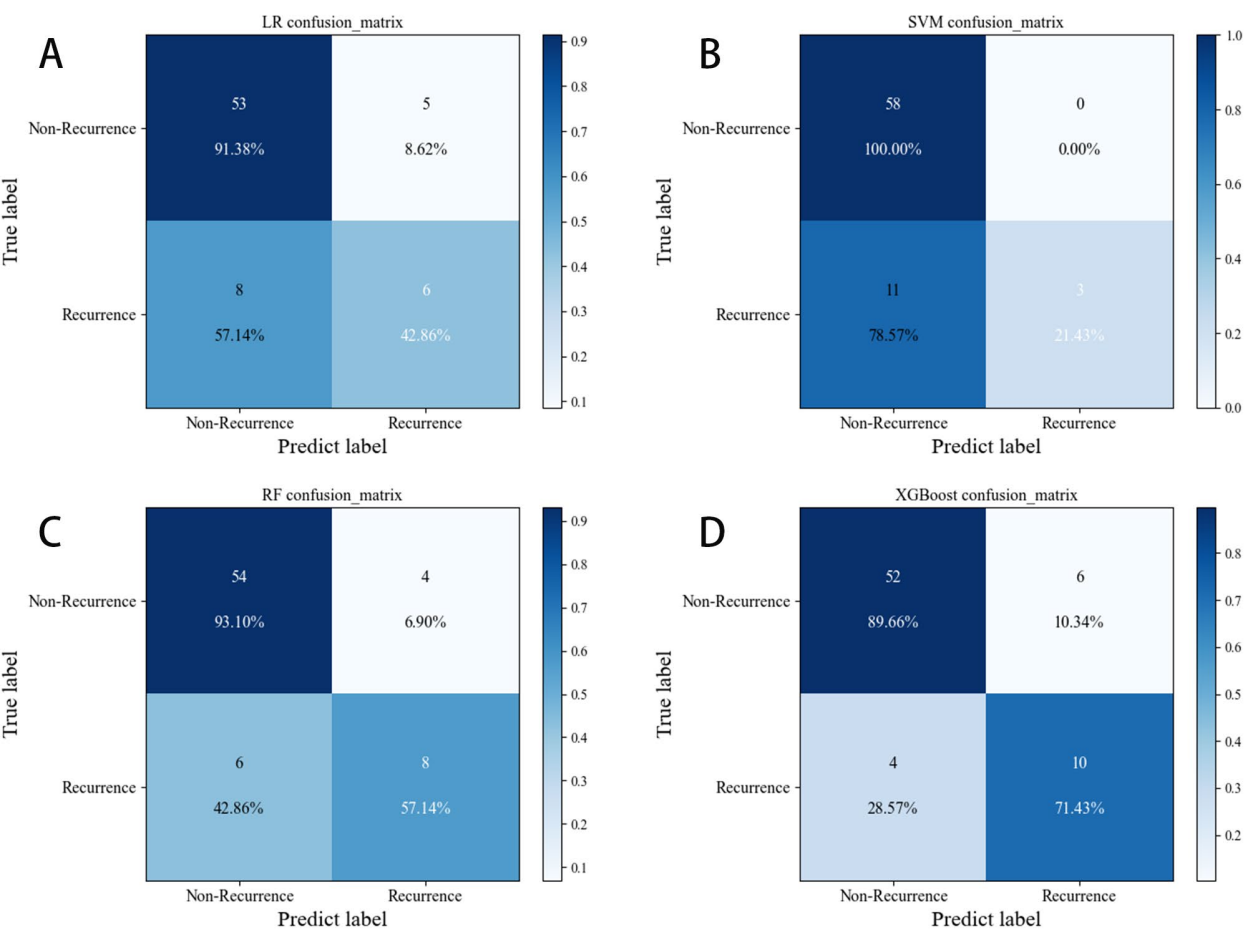
(**A**) Confusion matrix of the testing set in the logistic regression; (**B**) Confusion matrix of the testing set in SVM; (**C**) Confusion matrix of the testing set in RF; (**D**) Confusion matrix of the testing set in XGBoost

**Fig. 5:**
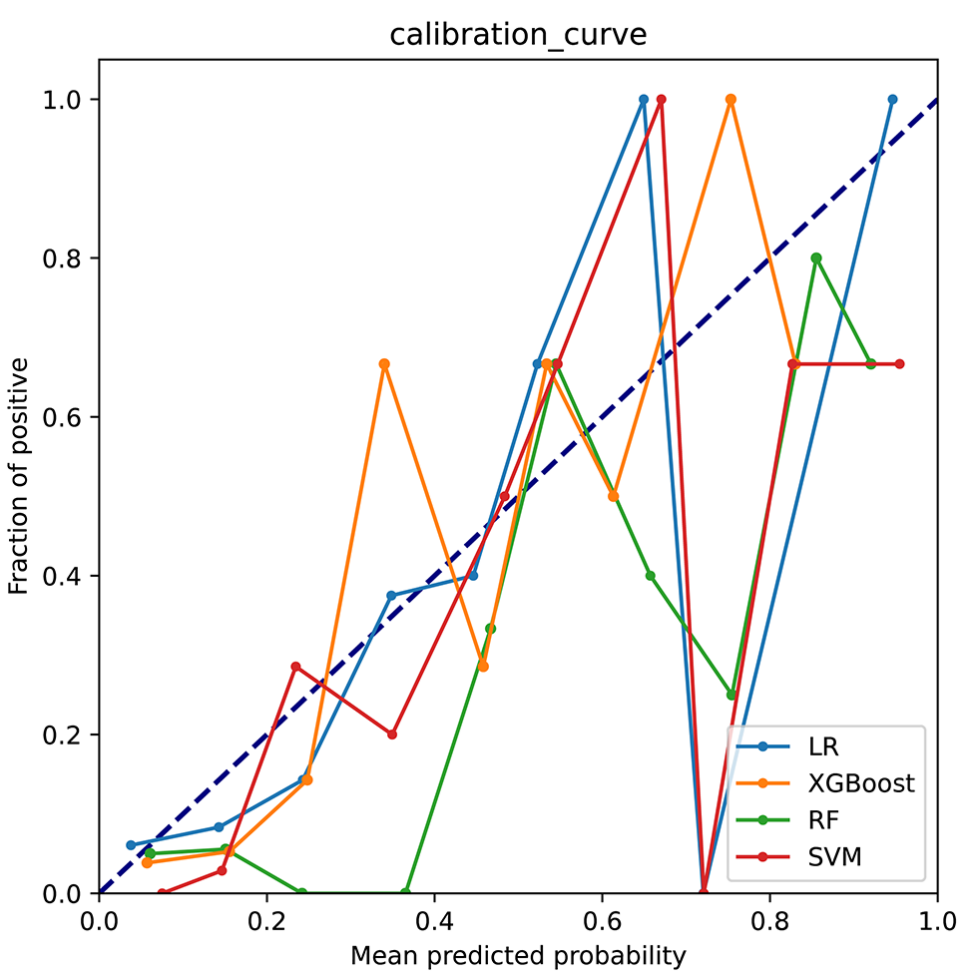
Calibration curves of the four models. The closer the curve is to the diagonal, the closer the predicted value is to the real value

A total of 359 patients were enrolled in this study, but only 61 patients relapsed after RFA. Therefore, it can be said that the sample distribution was unbalanced. To solve the problem of uneven sample distribution, we applied the synthetic minority oversampling technique (SMOTE) to balance the samples in this study. We also applied standardized methods to process the selected 6-dimensional variables before constructing the models. In addition, we simultaneously plotted a precision-recall (PR) curve to fully assess the model power. After adjusting the model parameters (Supplementary Table 1), we found that the logistic regression (LR), SVM, RF and XGBoost models performed well on the training set. As shown in Fig. 3A and Fig. 3B, on the training set, XGBoost had a better performance (AUC = 0.92, AP = 0.77) than RF (AUC = 0.91, P = 0.73), SVM (AUC = 0.82, AP = 0.61) and LR (AUC = 0.85, AP = 0.58). In addition, XGBoost also had a better performance (AUC = 0.87, AP = 0.75) than RF (AUC = 0.86, AP = 0.73), SVM (AUC = 0.78, AP = 0.60), and LR (AUC = 0.82, AP = 0.57) on the testing set (Fig. 3C and Fig. 3D). Figure 4 presents the confusion matrix of the four models in the testing set. Table 5 shows that the superior sensitivity and F1 score of XGBoost were 71.4% and 0.67, respectively. The XGBoost and RF prediction models had the same accuracy (86.1% and 86.1%). XGBoost had a lower specificity (89.7% and 93.1%) and precision (62.5% and 66.7%) than the RF model in predicting 12-month AF recurrence after RFA. Finally, the calibration curve of XGBoost in Fig. 5 was closer to the diagonal (y = x). In view of clinical practice, it is more important to identify as many patients as possible who relapse after RFA. Therefore, the XGBoost model has the best performance and clinical application of the four models.

**Table 5:** Model performance on the test set

Value	LR	SVM	RF	XGBoost
AUC	0.82	0.78	0.86	0.87
AP	0.57	0.60	0.73	0.75
Accuracy (%)	81.9	84.7	86.1	86.1
Specificity (%)	91.3	100.0	93.1	89.7
Sensitivity (%)	42.9	21.4	57.1	71.4
Precision (%)	54.5	100.0	66.7	62.5
F1 score	0.48	0.35	0.62	0.67

### Explanatory nature of model parameters

**Fig. 6:**
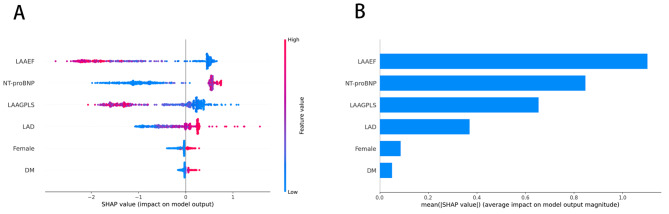
(**A**) The SHAP value of each variable in the XGBoost model. Each row represents a variable, and values closer to the top indicate a greater average SHAP value. The SHAP value represents the degree of influence of the variable on the results. One point in each variable represents a sample, and a deeper red colour indicates a greater characteristic value; (**B**) The average SHAP value of each variable; variables contribute more to the prediction model when their SHAP value is larger

To investigate the contribution of the parameters in predicting 12-month AF recurrence, we also applied a SHAP algorithm for the calculation and ranking of the contribution of each parameter; this is illustrated in Fig. 6A, in which LAAEF and LAAGPLS are shown to be negatively correlated with 12-month AF recurrence after RFA, while NT-proBNP, LAD, female sex and DM history are positively correlated with 12-month AF recurrence after RFA. As shown in Fig. 6B, the SHAP values of LAAEF, NT-proBNP, LAAGPLS and LAD were larger, indicating that they were important predictors of 12-month AF recurrence. Female sex and DM history had smaller SHAP values; thus, they made limited contributions to the model.

## Discussion

This study demonstrated the use of LAA functional parameters in predicting AF recurrence 12 months after RFA, of which LAAEF had the greatest application value, while the optimized LAA functional evaluation index LAAGPLS also had excellent application value. In addition, we combined the results of LAA functional parameters, cardiac global structural and functional parameters, and laboratory tests to construct four clinical prediction models for 12-month AF recurrence after RFA, of which the XGBoost model had the greatest value.

Changes in left atrial structure and function are essential in the development of AF. Several studies have linked left atrial indicators to recurrence of AF after RFA, even in patients undergoing cryoablation [15, 26, 27]. The majority of current studies revealed that left atrial enlargement was an independent predictor of recurrence after RFA [28], while scholars in some studies reported a U-shaped relationship between left atrial size and AF recurrence; that is, patients with small atria were also prone to AF recurrence [8]. In our study, left atrial size was positively correlated with AF recurrence 12 months after RFA. Heart failure is mainly characterized by changes such as decreased LVEF or increased NT-proBNP, and such patients are more likely to suffer from left atrial remodelling due to increased left atrial pressure [3, 29]. Our study found that an increase in NT-proBNP was an independent predictor of AF recurrence 12 months after RFA, which was consistent with previous studies [9, 30, 31]. Compared with research that proved that EDT could reflect cardiac function to some extent [32], the results of this study suggested a certain effect of EDT on predicting 12-month recurrence of AF.

Inconsistent results concerning the use of female sex as a predictor of recurrence after AF ablation have been reported. Some studies have shown a similar recurrence after RFA between females and males [33–35]. However, a recent large sample size study revealed that females have a higher recurrence rate after RFA (OR: 1.26, 95% CI: 1.15–1.38) [35]. In our study, female sex seemed to be associated with AF recurrence 12 months after RFA. However, it had a limited application value due to the small SHAP value. Previous studies also revealed that patients with DM had a greater risk of AF recurrence after RFA than those without DM [36, 37]. It appeared that including the variable of DM can increase the predictive power of our models. Since the variable had a minimal SHAP value, we can only state that it is of rather limited use in predicting the 12-month recurrence of AF.

As an accessory structure of the left atrium, the LAA has many trabecular muscles inside, and the myocardial thickness is thin, with a thickness of approximately 1 mm [38]. Some studies on the physiological roles of LAA have shown that 30% of the atrial natriuretic peptide was secreted by the LAA [39]. In addition, the LAA is also the origin of many atrial arrhythmias [40, 41]. In patients with AF, structural and functional changes also occur in the LAA, and LAA structural and functional parameters can predict the recurrence of AF after RFA [42, 43]. Tian et al. followed up on patients undergoing RFA for 19 months, finding a high clinical value of LAAEF in predicting the recurrence of AF with a hazard ratio of 0.790 (95% CI: 0.657–0.950) [20]. In our study, LAAEF was negatively correlated with the 12-month AF recurrence rate with the largest SHAP value, indicating that LAAEF had important clinical value in predicting 12-month recurrence of AF after RFA. New findings in myocardial strain in recent years have shown a high clinical value of left atrial strain in predicting recurrence of AF after RFA, but studies on the value of LAAGPLS remain scarce or have had nonsignificant results [21, 44]. In our study, the follow-up results of 359 patients revealed that LAAGPLS was an independent predictor of AF recurrence after RFA. Considering situations of complex LAA movement patterns, difficult strain measurement, and poor reproducibility, we defined LAAGPLS as the sum of the positive and negative absolute global peak strain values, which was more repeatable. Hence, we believe that the positive results of this study were related to our optimization of measurement methods.

At this stage, artificial intelligence algorithms are widely applied to solve clinical problems due to their ability to overcome the limitations of traditional linear relationships between dependent and independent variables. RF and XGBoost, in particular, as tree model-based integration algorithms, have shown high application value in clinical classification and regression problems [45, 46]. Labarbera et al. noted that a machine learning algorithm combining pulmonary vein morphology and clinical data was useful for predicting AF recurrence after RFA [47], but no known studies have focused on the predictive value of a machine learning model of the functional parameters of the left atrium and LAA. Our machine learning model combined clinical indicators and transoesophageal echocardiographic functional parameters, and the results indicated that the XGBoost algorithm had the best performance and could be used for accurate prediction of AF recurrence 12 months after RFA.

### Limitations

In this study, some limitations were identified. Since this study was conducted in a single centre, external validation was not possible. Second, in this study, the sample size was relatively small, with only 61 cases in the recurrence group. Third, the follow-up time of our study was 12 months; thus, there was an absence of long-term follow-up on AF recurrence after RFA. Last, many clinical parameters, such as AF duration, AF burden, and ECG parameters, such as P-wave duration, were not studied in our research. In the future, a larger sample size and more clinical parameters will be needed to improve the efficiency of these models, and an external dataset will also be needed to validate the findings of our model. In addition, we will continue exploring the application of deep learning in predicting AF recurrence.

## Conclusion

Due to the high recurrence rate of RFA in AF patients, accurate prediction of the recurrence risk after RFA has important clinical implications. In our study, we selected 6 variables and compared 4 machine learning methods to predict the recurrence of AF 12 months after RFA, and XGBoost had the greatest performance (AUC = 0.87, AP = 0.56, accuracy = 86.1%, specificity = 89.7%, precision = 62.5%, F1 score = 0.67) in the testing set. To obtain the weight of the importance of variables in the XGBoost model, we used the variable importance indicator. The results showed that LAA functional parameters, including LAAEF and LAAGPLS, have important clinical value in predicting AF recurrence after RFA.

### Electronic supplementary material

Below is the link to the electronic supplementary material.

Supplementary Material 1

## Data Availability

Data supporting the findings of this study can be obtained from the corresponding authors upon reasonable request.
