# Supplementary material for: An effective prediction model based on XGBoost for the 12-month recurrence of AF patients after RFA
Source: BMC Cardiovasc Disord. 2023 Nov 16;23:561. doi: 10.1186/s12872-023-03599-9 (PMC10655386; doi:10.1186/s12872-023-03599-9)
Supplement: Supplementary file 1 — Supplementary Material 1 [file 12872_2023_3599_MOESM1_ESM.docx]

model parameters

| algorithm | Parameters |
| --- | --- |
| XGBoost | XGBClassifier(base_score=0.5, booster='gbtree', colsample_bylevel=1, colsample_bynode=1, colsample_bytree=0.8, gamma=1, learning_rate=0.01, max_delta_step=0, max_depth=4, min_child_weight=1.5, missing=None, n_estimators=18, n_jobs=1, nthread=None, objective='binary:logistic', random_state=27,  reg_alpha=0.1, reg_lambda=1, scale_pos_weight=5, seed=None, silent=None, subsample=0.6, verbosity=1) |
| LogisticRegression | LogisticRegression(C=0.3, class_weight=None, dual=False, fit_intercept=True, intercept_scaling=1, l1_ratio=None, max_iter=100, multi_class='auto', n_jobs=None, penalty='l2', random_state=None, solver='lbfgs', tol=0.0001, verbose=0, warm_start=False) |
| SVM | SVC(C=1.1, kernel=’rbf’, degree=3, gamma=’scale’, coef0=0, shriking=True, probability=True, tol=1e-3, cache_size=200, class_weight=None, verbose=False, max_iter=-1, decision_function_shape=’ovr’, break_ties=False, random_state=None) |
| RandomForest | RandomForestClassifier  (n_estimators: = 15,  criterion: = "gini",  max_depth: =3,  min_samples_split: = 2,  min_samples_leaf: = 1,  min_weight_fraction_leaf: = 0.0,  max_features: = "auto",  max_leaf_nodes: = None,  min_impurity_decrease: = 0.0,  bootstrap: = True,  oob_score: = False,  n_jobs: = None,  random_state: = 14,  verbose: = 0,  warm_start: = False,  class_weight: = None,  ccp_alpha: = 0.0,  max_samples: = None) |
